# The Glucocorticoid Resistance Syndrome. Two Cases of a Novel Pathogenic Variant in the Glucocorticoid Receptor Gene

**DOI:** 10.1210/jcemcr/luad153

**Published:** 2024-01-02

**Authors:** Sílvia Mauri, Javier Nieto-Moragas, María Obón, Josep Oriola

**Affiliations:** Diabetes, Endocrinology and Nutrition Unit, Hospital Universitari de Girona Dr. Josep Trueta, 17007 Girona, Spain; Clinical Genetics Department, Girona Territorial Laboratory, Parc Hospitalari Martí Julià, 17190 Salt (Girona), Spain; Clinical Genetics Department, Girona Territorial Laboratory, Parc Hospitalari Martí Julià, 17190 Salt (Girona), Spain; Biochemistry and Molecular Genetics Department, CDB, Hospital Clínic de Barcelona, Faculty of Medicine, University of Barcelona, 08036 Barcelona, Spain

**Keywords:** glucocorticoid receptor gene (NR3C1), glucocorticoid resistance syndrome

## Abstract

Glucocorticoid resistance syndrome is a rare genetic condition characterized by generalized or partial target-tissue insensitivity to glucocorticoids and a consequent hyperactivation of the hypothalamic-pituitary-adrenal axis. Clinical manifestations may include mineralocorticoid and/or androgen excess without manifestations of Cushing syndrome. At a cellular level, glucocorticoid actions are mediated by the nuclear glucocorticoid receptor encoded by the *NR3C1* gene. To date, only 33 glucocorticoid receptor loss-of-function pathogenic variants have been associated with glucocorticoid resistance syndrome. The *NR3C1* gene has 2 known disease-causing mechanisms: haploinsufficiency and negative dominance. We describe a mother and her son with a mild hyperandrogenic phenotype and a novel genetic variant of the *NR3C1* gene predicting a truncated protein and causing glucocorticoid resistance syndrome. To date, no accurate genotype-phenotype correlation has been found.

## Introduction

Glucocorticoids are steroid hormones synthesized in the zona fasciculata of the adrenal cortex and act as the end products of the hypothalamic-pituitary-adrenal axis. Glucocorticoids have a wide range of physiological processes associated with growth, inflammatory processes, tolerance to stress, carbohydrate-protein-fat and bone metabolism, and sexual development. They work by binding to the human glucocorticoid receptor (hGR), a ubiquitously expressed protein located in the nuclear region of target cells. Both synthetic and natural glucocorticoids can bind to the hGR [1].

Pathogenic variants in the hGR produce glucocorticoid resistance syndrome (GRS), also named Chrousos syndrome. GRS is an inherited condition with an autosomal dominant pattern. The hGR is encoded by the *NR3C1* (NM_000176.3) gene, located on chromosome 5 (5q31.3) and containing 9 exons. To date, 33 *NR3C1* loss-of-function pathogenic variants have been associated with GRS (OMIM#615962). Among these variants, 23 are missense, 5 frameshift, 4 nonsense, and 1 affecting the splice site in exon-intron 6 [2‐5].

GRS is an extremely rare endocrine disorder that affects tissues expressing the hGR and is characterized by a generalized or partial decreased sensitivity to glucocorticoids due to a defective hGR in both the hypothalamus and pituitary gland that results in an impaired cortisol negative feedback loop, driving hypersecretion of corticotropin-releasing hormone, arginine vasopressin, and ACTH. The increased plasma ACTH concentrations lead to stimulation of the adrenal cortex with release of cortisol, adrenal androgens (androstenedione, dehydroepiandrosterone), mineralocorticoid steroids (corticosterone and deoxycorticosterone), and adrenal hyperplasia.

Patients with GRS may have biochemical hypercortisolism without Cushing disease symptoms. The clinical phenotype is diverse, ranging from asymptomatic cases to severe mineralocorticoid (hypertension, hypokalaemia, and alkalosis) and/or androgen excess (hirsutism, acne, male-pattern hair loss, precocious puberty, amenorrhoea or hypo fertility, and menstrual irregularities in females and oligospermia in males). Hypercortisolism may be the cause of patient persistent fatigue while increased corticotropin-releasing hormone and arginine vasopressin secretion may lead to anxiety and sadness in these patients.

Diagnostic evaluation of the GRS includes a complete medical history with an emphasis on the absence of symptoms and signs of hypercortisolism, determination of morning serum cortisol under fasting conditions and after dexamethasone administration, 24-hour urinary free cortisol (UFC), plasma ACTH, serum aldosterone, plasma renin activity, and androgens. The *NR3C1* sequencing is required for diagnostic confirmation.

## Case Presentation

### Case 1

A 49-year-old female was evaluated at the endocrine department because of hirsutism, childhood acne, and irregular menstrual cycles. She had conceived through in vitro fertilization and was being treated for anxiety. She had gone through several rounds of hair removal sessions. No skin atrophy, buffalo hump, purple striae, myopathy, or truncal obesity were present. Her body mass index was 22.6 kg/m2, and blood pressure, potassium levels, and glucose homeostasis were normal.

### Case 2

We also analyzed her 17-year-old son, who presented precocious puberty with severe hirsutism, acne, short stature (height 165 cm, body mass index 20 kg/m2), and advanced bone maturation. His blood pressure was 128/70 mmHg. Laboratory findings (Table 1) also revealed increased ACTH and 24-hour UFC and resistance of the hypothalamic-pituitary-adrenal axis to low-dose dexamethasone suppression. Genetic analysis revealed he also was a carrier of the mother's variant.

**Table 1.** Laboratory findings

	Mother	Son	Reference Range
Cortisol	**26.31 mcg /dL** **(725.79 nmol/L)**	17.25 mcg /dL (475.86 nmol/L)	6-19.4 mcg /dL (165.52-535.17 nmol/L)
UFC	**455.8 mcg /24h** **(1256.0 nmol/24h)**	**165.8 mcg /24h** **(456.9 nmol/24h)**	12.8-82.5 mcg /24h (35.3-227.3 nmol/24h)
ACTH	**184.4 pg/mL** **(40.6 pmol/L)**	**56.1 pg/mL** **(12.4 pmol/L)**	4.7-48.8 pg/mL (1.0-10.8 pmol/L)
Cortisol after LDDST	**4.5 mcg /dL** **(124.1 nmol/L)**	**5.5 mcg /dL** **(151.7 nmol/L)**	< 1.8 mcg /dL (<49.7 nmol/L)
DHEA-S	1.4 mcg/mL (3.8 µmol/L)	5.1 mcg /mL (13.7 µmol/L)	1.5-7.7 mcg /mL (4.1-20.9 µmol/L)
Androstenedione	2.5 mcg/L (8.7 nmol/L)	**3.8 mcg/L** **(13.3 nmol/L)**	(F): 0.3-3.7 mcg/L (1.1-13.3 nmol/L) (M) 0.6-3.1 mcg/L (2.1-10.8 nmol/L)
Testosterone	0.32 ng/ml (1.11 nmol/L)	6.80 ng/ml (23.58 nmol/L)	(F) 0.06-0.82 ng/ml (0.21-2.84 nmol/L) (M) 2.8-8 (9.71-27.74 nmol/L)
Potassium	4.4 mmol/L	4.7 mmol/L	3.5-5.1 mmol/L
Renin	12.88 mcUI/ml	33.7 mcUI/ml	2.8-46.1 mcUI/ml
Aldosterone	6.53 pg/mL (0.0181 nmol/L)	4.56 pg/mL (0.0127 nmol/L)	1.76-39.2 pg/mL (0.0049-0.1088 nmol/L)

Bold values are those outside reference range.

Abbreviations: DHEAS, dehydroepiandrosterone sulphate; LDDST, low-dose dexamethasone suppression test; UFC, urinary free cortisol.

Some possible differential diagnosis may include:

- Mild forms of Cushing syndrome, in which hypercortisolism is accompanied by normal or mildly elevated ACTH concentrations, preserved circadian pattern of ACTH and cortisol secretion, and lack of cortisol supression by dexamethasone.
- Pseudocushing states, such as genealized anxiety disorder or melancholic depression.
- Conditions associated with elevated serum concentrations of cortisol-binding globulin.
- Other causes of hyperandrogenism or virilization such as polycystic ovarian syndrome in females or congenital adrenal hypeplasia.

## Diagnostic assessment

Hormone laboratory findings are shown in Table 1 and revealed elevated morning serum cortisol levels 26.31 mcg/dL [725.79 nmol/L; reference range values: 6-19.4 mcg/dL (165.52–535.17 nmol/L)] with plasma ACTH levels 184.4 pg/mL [40.6 pmol/L; reference range values: 4.7-48.8 pg/mL (1.0–10.8 pmol/L)], and UFC concentrations 455.8 mcg/24 hours [1256 nmol/L; reference range values 12.8-82.5 mcg/24 hours (35.3–227.3 nmol/L)] above the upper limit. An overnight dexamethasone suppression test with 1 mg of dexamethasone revealed insufficient suppression of morning serum cortisol 4.5 mcg/dL (124 nmol/L) <1.8 mcg/dL (< 49.7 nmol/L). Serum levels of dehydroepiandrosterone sulphate, testosterone, aldosterone, 17 OH progesterone, and androstenedione were all within normal limits. Neither adrenal computed tomography scan, pituitary magnetic resonance imaging, or bone mineral density via dual-energy x-ray absorptiometry revealed any abnormalities.

## Treatment

Treatment aims to reduce excessive endogenous ACTH secretion, especially in patients with significant hGR action impairment, by administering increasing doses of synthetic glucocorticoids.

## Outcome and Follow-up

**Figure 1.**
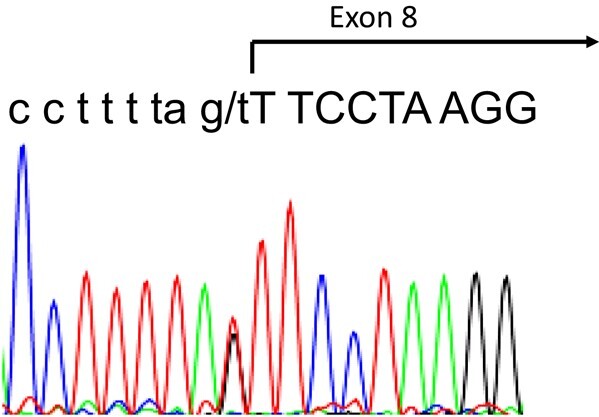
Electropherogram showing the c.2024-1G > T variant in heterozygosis, in the NR3C1 gene in index case.

**Figure 2.**
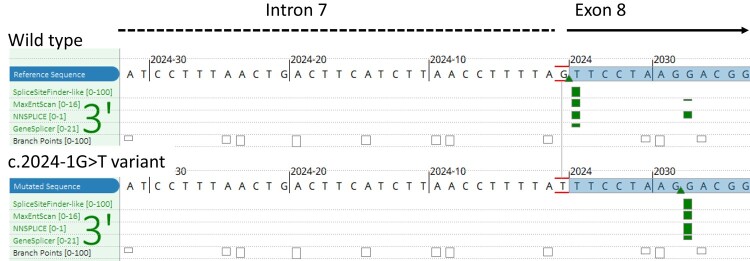
Different in silico study tools predict the skipping of the first eight coding nucleotides of exon 8 when the c.2024-1G > T variant is present.

Following genetic counselling and written informed consent, polymerase chain reaction amplification and Sanger sequencing were used to analyze the *NR3C1* gene, and the c.2024-1G > T variant was found in heterozygosis. This variant is located at the end of intron 7 (Fig. 1) and predicts the skipping of the first 8 coding nucleotides of exon 8 (Fig. 2).

## Discussion

We report a second splice-site variant (c.2024-1G > T) of the *NR3C1* gene. In this case, this change occurs in the nuclear localization signal 2, predicting a protein frameshift from codon 675, p.(Val675Glyfs*10) and, as a consequence, a premature terminal codon [6].

The *NR3C1* gene has known 2 disease-causing mechanisms: haploinsufficiency and negative dominance. Haploinsufficiency can be caused by changes that result in truncated proteins but also by missense variants, and negative dominance is mainly caused by missense changes. Taking both mechanisms into account, it would be noteworthy to check if variants predicted to produce a frameshift correlate with a mild clinical phenotype (as observed in our case) because there is practically no interference with the normal product. When more variants predicting frameshift or missense changes are described, we could envisage this possible genotype-phenotype correlation.

## Learning Points

- GRS is a rare endocrine disorder due to mutations in the hGR encoded by the NR3C1 gene.
- The clinical spectrum of GRS is broad, ranging from most severe to mild features of mineralocorticoid and/or androgen excess.
- Laboratory findings present normal or elevated plasma ACTH concentrations with increased 24-hour UFC excretion and inadequate suppression of morning serum cortisol to low-dose dexamethasone suppression test.
- To date, 33 NR3C1 loss-of-function pathogenic variants have been associated with GRS.

## Contributors

All authors made individual contributions to authorship. S.M. was involved in the management of the patient and manuscript submission. J.N. and M.O. were involved in hormone laboratory diagnosis and genetic counseling. J.O. was involved in genetic findings. All authors reviewed and approved the final draft.

## Data Availability

Original data generated and analyzed during this study are included in this published article.

## References

[1] Nicolaides NC, Charmandari E. Primary generalized glucocorticoid resistance and hypersensitivity syndromes: a 2021 update. Int J Mol Sci. 2021;22(19):10839. https://www.mdpi.com/1422-0067/22/19/10839 34639183 10.3390/ijms221910839 PMC8509180

[2] Molnár Á, Patócs A, Likó I, et al An unexpected, mild phenotype of glucocorticoid resistance associated with glucocorticoid receptor gene mutation case report and review of the literature. BMC Med Genet. 2018;19(1):37. 29510671 10.1186/s12881-018-0552-6 PMC5840839

[3] Lin L, Wu X, Hou Y, Zheng F, Xu R. A novel mutation in the glucocorticoid receptor gene causing resistant hypertension. Am J Hypertens. 2019;32(11):1126‐1128. 31414133 10.1093/ajh/hpz137

[4] Vitellius G, Lombes M. Genetics in endocrinology: glucocorticoid resistance syndrome. Eur J Endocrinol. 2020;182(2):R15‐R27. https://eje.bioscientifica.com/view/journals/eje/182/2/EJE-19-0811.xm 31995340 10.1530/EJE-19-0811

[5] Charmandari E, Kino T, Chrousos GP. Familial/sporadic glucocorticoid resistance: clinical phenotype and molecular mechanisms. *Ann NY Ac Sci*. 2004:168-181. 10.1196/annals.1321.014 15265781

[6] Richards S, Aziz N, Bale S, et al Standards and guidelines for the interpretation of sequence variants: a joint consensus recommendation of the American college of medical genetics and genomics and the association for molecular pathology. Genet Med. 2015;17(5):405‐424. 25741868 10.1038/gim.2015.30 PMC4544753

